# Mapping and identification of molecular markers for the *Pc96* gene conferring resistance to crown rust in oat

**DOI:** 10.1371/journal.pone.0283769

**Published:** 2023-04-06

**Authors:** Sidrat Abdullah, Tyler Gordon, Belayneh Admassu Yimer, Edyta Paczos-Grzęda, Stephen A. Harrison, James G. Menzies, Kathy Esvelt Klos

**Affiliations:** 1 Oak Ridge Institute for Science and Education (ORISE) Research Participant, Small Grains and Potato Germplasm Research Unit, Agricultural Research Service, United States Department of Agriculture, Aberdeen, ID, United States of America; 2 Small Grains and Potato Germplasm Research Unit, Agricultural Research Service, United States Department of Agriculture, Aberdeen, ID, United States of America; 3 Department of Plant Sciences, University of Idaho, Aberdeen, ID, United States of America; 4 Institute of Plant Genetics, Breeding and Biotechnology, University of Life Sciences, Akademicka, Lublin, Poland; 5 School of Plant, Environmental and Soil Sciences, Louisiana State University Agricultural Centre, Baton Rouge, Louisiana, United States of America; 6 Morden Research and Development Centre, Agriculture and Agri-Food Canada, Morden, Manitoba, Canada; Murdoch University, AUSTRALIA

## Abstract

Oat crown rust caused by *Puccinia coronata* f. sp. *avenae P*. *Syd*. & *Syd* (*Pca*) is a major constraint to oat (*Avena sativa* L.) production in many parts of the globe. The objectives of this study were to locate *Pc96* on the oat consensus map and to develop SNP markers linked to *Pc96* for use in marker-assisted selection. SNP loci linked to the crown rust resistance gene *Pc96* were identified by linkage analysis and PACE assays were developed for marker-assisted selection in breeding programs. *Pc96* is a race-specific crown rust resistance gene originating from cultivated oat that has been deployed in North American oat breeding programs. *Pc96* was mapped in a recombinant inbred line population (n = 122) developed from a cross between the oat crown rust differential known to carry *Pc96* and the differential line carrying *Pc54*. A single resistance locus was identified on chromosome 7D between 48.3 and 91.2 cM. The resistance locus and linked SNPs were validated in two additional biparental populations, Ajay **×** Pc96 (F_2:3_, n = 139) and Pc96 **×** Kasztan (F_2:3_, n = 168). Based on all populations, the most probable location of the oat crown rust resistance gene *Pc96* on the oat consensus map was on chromosome 7D approximately at 87.3 cM. In the Ajay **×** Pc96 population, a second unlinked resistance gene was contributed by the Pc96 differential line, which mapped to chromosome 6C at 75.5 cM. A haplotype of nine linked SNPs predicted the absence of *Pc96* in a diverse group of 144 oat germplasm. SNPs that are closely linked to the *Pc96* gene may be beneficial as PCR-based molecular markers in marker-assisted selection.

## Introduction

Oat (*Avena sativa* L.) is a widely grown cereal, used for grain, forage, and straw production [1]. Oat is of special interest from a human health perspective because of the presence of β-glucans, which have been shown to lower blood cholesterol levels and the risk of cardiovascular diseases [2]. The primary disease limiting oat production globally is crown rust caused by *Puccinia coronata* f. sp. *avenae P*. *Syd*. & *Syd* (*Pca*). This economically important disease occurs in most areas where oat cultivars and wild oats are grown [3, 4]. Average oat yield losses due to crown rust generally vary from 1% to 5% per year in North America, but more severe losses are routinely reported [3, 5] and can be much higher in individual environments. Crown rust management strategies include early planting, scouting and removal of the alternate host, Buckthorn (*Rhamnus* spp.), fungicide application and genetic resistance [3, 6–8].

Single (*Pc*) gene resistance can be effective against specific *Pca* races and is easy to incorporate into new cultivars. For this reason, incorporation of single gene resistance into cultivated oat germplasm has been a common strategy for managing oat crown rust [9–11]. Oat crown rust qualitative resistance genes with known genomic locations include *Pc38* [12], *Pc39* [13, 14], *Pc45* [15], *Pc48* [16], *Pc50-5* [17], *Pc53* [18], *Pc54* [19], *Pc58*a [20, 21], *Pc68* [22], *Pc71* [23], *Pc91* [24], *Pc94* [25] and *Pc98* [26]. Unfortunately, single gene resistance is typically only effective for a few years due to rapid changes in *Pca* virulence [27–31]. Oat crown rust qualitative resistance is controlled by a single major gene and pyramiding multiple major genes in a single cultivar has been proposed as a method to provide long lasting, effective resistance [7]. Developing molecular markers that are closely linked with major genes can allow for reliable introgression of multiple major genes in a marker assisted selection (MAS) breeding program.

In an ongoing effort to identify the genomic locations of all known *Pc* genes, the purpose of this study was to genetically map the location of the *Pc96* gene. *Pc96* was obtained from the oat accession MG 85039, which was developed by the National Research Council Germplasm Institute, Bari, Italy. The *Pc96* single gene differential line has the pedigree: Makuru × MG 85039 [32]. Although *Pca* isolates with virulence to *Pc96* have been documented [13, 31], this gene has provided a moderate level of resistance against a diverse group of *Pca* isolates in the Matt Moore Buckthorn Nursery in St. Paul, MN since 2016 (T. Gordon, Personal communication) and in Canada since 2010 [33]. The Buckthorn nursery consists of oat lines planted among the alternate host of crown rust, *Rhamnus cathartica* L. and has been in continuous use for oat crown rust research since the 1950s [34]. Therefore, performance of *Pc* genes within this nursery is impacted by a sexually recombining, highly diverse crown rust population.

The objectives of this study were to: i) identify the chromosome region linked with *Pc96*, ii) develop molecular markers closely linked with *Pc96*, and iii) validate the genetic position and markers closely linked with *Pc96* in two additional biparental populations and a panel of diverse oat germplasm.

## Material and methods

### Plant material

A recombinant inbred line (RIL) population (Pc54 × Pc96) of 122 F_5_-derived RILs was developed from a cross between the oat crown rust differential known to carry *Pc96* (Makuru × MG 85039) originally identified by Chong and Brown [32] and the oat differential known to carry *Pc54* (Pendek*2 × CAV1832) described by Martens et al. [35]. Two biparental populations were developed for gene validation: one was comprised of 139 F_3_-derived lines from a cross between the susceptible cultivar ‘Ajay’ (74AB1952 × 74AB2608) and the Pc96 oat crown rust differential (Ajay × Pc96). The second population consisted of 169 F_2_ lines and 168 F_2:3_ lines from a cross between the *Pc96* differential line and the susceptible cultivar ‘Kasztan’ (Dawid × CHD 1685/84). Allele frequencies of linked markers in the Collaborative Oat Research Enterprise (CORE) association mapping panel [36] were used to evaluate marker performance in 114 lines of the CORE with a susceptible reaction to all 10 isolates used in the Esvelt Klos et al. [37] study and with severity ratings >30% at all field location years. Those, along with 30 *Pc* differential lines, were defined as non-carriers of *Pc96* (Esvelt Klos et al. [37]; Source: http://triticeaetoolbox.org/oat/).

#### Phenotyping

*Controlled environment screening*. Seedlings of all three populations were evaluated in a growth chamber for their reaction to crown rust as previously described Yimer et al. [18]. Seedling tests were conducted at USDA-ARS, Aberdeen, ID; except the Pc96 × Kasztan population which was tested at the Morden Research and Development Centre, Agriculture and Agri-Food Canada between December 2018 and July 2019. *Pc* genes that were effective against each *Pca* isolate used in these experiments are shown in S1 Table [33, 38]. Parents were included as internal checks spaced every 50 entries. Approximately two weeks after planting, when seedlings were at the two-leaf stage, they were inoculated with uredinia suspended in Soltrol 170 isoparaffin oil (Chevron Phillips, The Woodlands, TX) and adjusted to a concentration of 2.0 x 10^5^ mL^-1^. Seedlings were grown in a growth chamber set at 20°C with a 14h photoperiod and assessed approximately 14 days post inoculation (dpi) for infection type based on scoring guide developed [38, 39] where infection type (IT) can range between 0 = no uredia, immune and 4 = large uredia; very susceptible. ITs of 0 to 2+ were considered resistant while 3 to 4 were considered susceptible [38].

About 4 seedlings from each of the 122 RILs were evaluated against *Pca* race NQBK where the *Pc54* differential had an IT of ‘1+’ and the *Pc96* differential had an IT of ‘0;’. The IT reactions were converted to binary scores where the susceptible parent reaction (or *Pc54*) was coded as ‘0’ and the reaction consistent with presence of *Pc96* was coded as ‘2’. *Pc96* carrier status was confirmed by evaluating four additional seedlings from the F_4:5_ generation. Individuals that did not have IT score present in the F_5_ generation were listed as missing. At least 20 seedlings from each F_2:3_ family were evaluated for the Ajay *×* Pc96 and Pc96 *×* Kasztan populations. The Ajay *×* Pc96 (n = 139) population was tested against *Pca* race MBTG which produced an IT of ‘3;’ or ‘4;’ with large uredia on Ajay, and an IT of ‘0;’ on the *Pc96* differential. The F_2_ (n = 169) and F_2:3_ (n = 168) families of the (Pc96 *×* Kasztan) population was screened with *Pca* race BRBG-94 which produced an IT of ‘4’ with large uredia on susceptible parent Kasztan, and an IT of ‘0’ on the resistant *Pc96* parent.

*Field screening*. Crown rust disease screening with 122 RILs of the Pc54 × Pc96 mapping population and the *Pc54* and *Pc96* parents was carried out in the Louisiana State University crown rust nursery in Baton Rouge, LA. Data was obtained from 107 RILs of the 122 field tested lines. Each RIL was planted in a nonreplicated meter-long row with 38 cm between rows, along with three replicated rows of parents. Crown rust was allowed to naturally infect the crown rust nursery and the population was evaluated on April 20, 2018 when lines were at the flag leaf stage [40]. Infection reaction (IR) of each RIL and of the parents was scored as resistant (R), moderately resistant (MR), moderately susceptible (MS) and susceptible (S), then these ratings were converted to a numerical scale where S = 1 and R = 0.2 [41].

### Genotyping and SNP marker development

The Pc54 *×* Pc96 and Ajay *×* Pc96 populations were genotyped from gDNA using the 6K Illumina Infinium iSelect oat SNP chip at the Cereal Crops Research Unit of ARS-USDA in Fargo, ND. SNPs were called automatically using the Genome Studio 2.0 DBSCAN procedure and were manually assessed for call accuracy based on instructions from the manufacturer (Illumina, San Diego, CA, 2016). Genotyping in the CORE and *Pc* differential lines were as previously described by Esvelt Klos, et al. [36]. Genotype calls for markers of interest on CORE lines were obtained from the T3 database (https://oat.triticeaetoolbox.org/) except where the quality control process described by Esvelt Klos, et al. [36] resulted in elimination of a marker of specific interest to this study. The Pc96 × Kazstan (F_2:3_) population was genotyped using SNP assays designed from Illumina SNP sequences with the following protocol: Competing SNP allele assays were designed and run according to instructions provided by the manufacturer of PACE genotyping master mix, 3CR Bioscience (Harlow, UK). Briefly, each SNP assay was designed with two allele-specific forward primers and one common reverse primer for each putative SNP. SNP assay reactions were prepared in a final volume of 10 μl which was comprised of 3 μl of genomic DNA (50 ng μl^−1^), 5 μl of 2×PACE reaction mix (StdRox), and 0.14 μl of primer mix (including 12 μl of each forward primer and 30 μl common primer) adjusted with water. The PCR protocol was run on a CFX96 (BioRad, Hercules, CA) with an initial denaturation for 15 min at 94° for, 10 touchdown cycles of 94°C for 20 s, 65°C for 60 s (dropping down by 0.6°C per cycle), and 30 cycles of 94°C for 20s, followed by extension at 55°C for 60 s and a plate read at 25°C after 60 s.

### Genetic map construction and statistical analysis

JMP Genomics v. 10.0 (Cary, NC) was used for all statistical analyses. Markers were removed from the F_5_-derived population (Pc54 × Pc96) when they had a missing data for markers (>10%), lines missing (>20%) and heterozygosity (>12%). The same procedure was followed for marker QC in Ajay × Pc96 (F_2:3_) except that the expected heterozygosity percentage was 6 to 40%.

Linkage maps were generated from segregating markers in order to calculate the genetic distance of the *Pc96* locus from nearby SNPs. Prior to linkage mapping, SNP markers were placed to chromosomes using the PepsiCo sequence ("*Avena sativa*–PepsiCo v1/Consensus_2018/Infinium 2020, https://oat.triticeaetoolbox.org/”). Markers were removed that were not segregating with others placed to the same chromosome. Initially, during linkage map construction distorted markers (P<0.001) were omitted from the analysis. Markers were also removed when their inclusion in the linkage group produced gaps that increased chromosomal length beyond ~250 cM. Linkage map order was determined by multidimensional scaling and the resistance loci were calculated in centiMorgans (cM) using the Kosambi map function [42]. Comparative maps were drawn using MapChart v. 2.1 [43]. Seedling resistance was placed to the linkage maps using the same methods as above.

The χ^2^ analysis of the Ajay × Pc96 populations suggested segregation for two resistance genes. Therefore, QTL analysis in the entire population was used to estimate the chromosomal location of each *R* gene. A minimum logarithm of odds (LOD) score of 3.4 for Ajay *×* Pc96 was determined after running a permutation test at alpha = 0.05. Markers on those chromosomes were used to define sub-populations (n = 112 for *Pc_MBTG_6C* and n = 91 for *Pc96*) segregating at one gene while fixed for the susceptible allele at the other. Linkage analysis was performed within these sub-populations as described above.

SNP assays were designed that are on the PepsiCo sequence on the right chromosome (7D) and used to validate markers associated with resistance in the *Pc96* × Kasztan population (S2 Table). Markers that distinguished the parents, *Pc96*, and Kasztan, into two different clusters were evaluated. Markers were also evaluated on other parents Pc54 and Ajay to check the efficiency of the markers that were developed from different populations. SNP markers anchored on chromosome 7D were genotyped in the Pc96 × Kasztan population to map *Pc96* using linkage analysis.

Multiple Interval Mapping (MIM) described by Kao et al. [44] was used to identify quantitative trait loci (QTL) associated with field (F_5:6_, n = 107) crown rust resistance in the Pc54 × Pc96 population. A forward search for QTL main effects was run to locate peak QTL positions and to detect possible epistatic-effect QTL interactions. A permutation test was performed with 1000 random reshuffles of observations, as recommended [45] to determine the statistical significance threshold. A minimum logarithm of odds (LOD) score of 3.0 for Pc54 *×* Pc96 was determined after running a permutation test at alpha = 0.05.

A Pearson’s Chi-squared (χ^2^) goodness of fit test was used to estimate the number of genes segregating within each population.

## Results

### Crown rust reaction and inheritance of resistance

**Fig 1:**
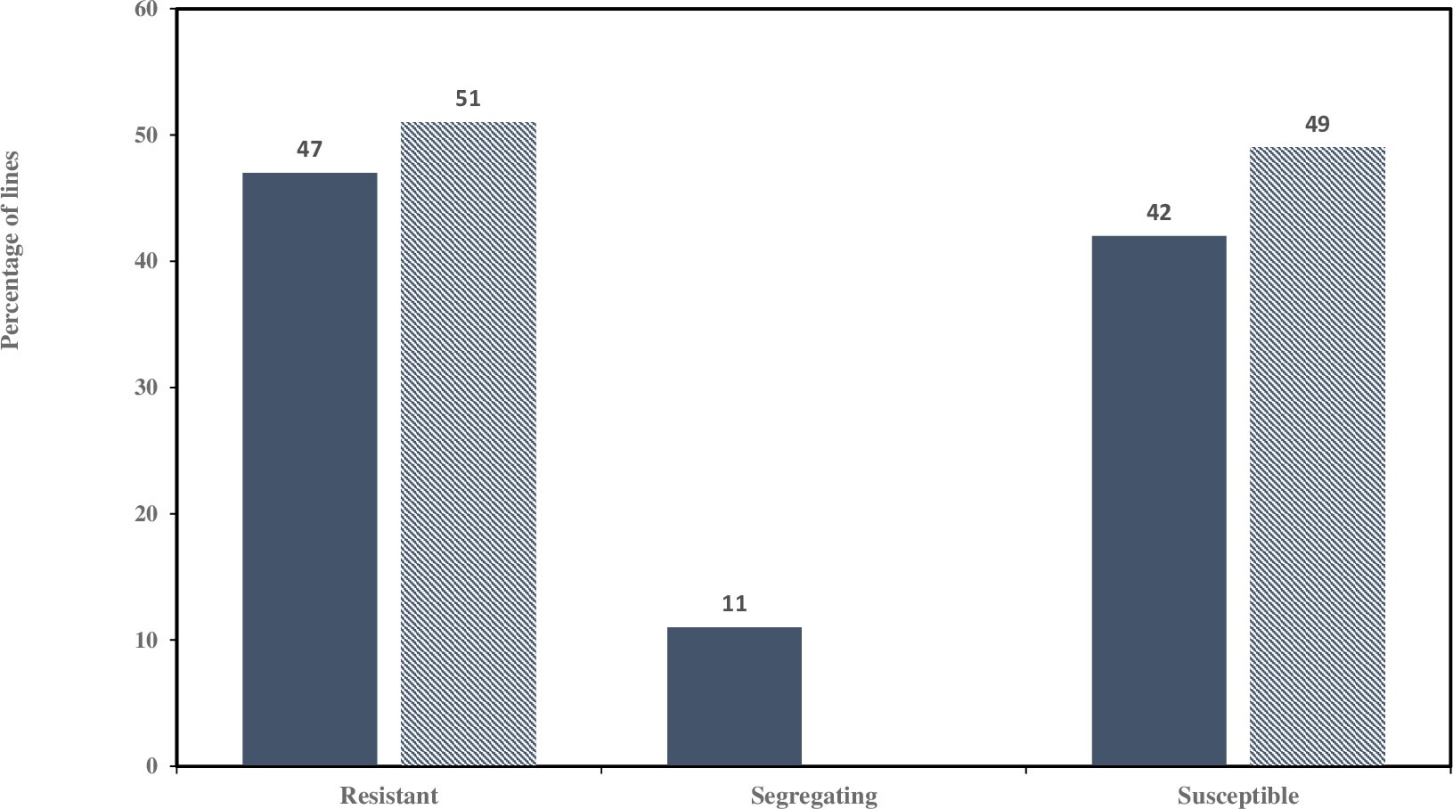
Distribution of crown rust phenotypes of the Pc54 × Pc96 population from greenhouse and field phenotyping at the seedling (solid bars) and flag leaf stages (dashed bars) respectively.

Seedling stage reactions of the *Pc96* differential line were resistant in all tests with an IT of ‘0’ or ‘0;’ to races NQBK, MBTG and BRBG-94, respectively. The *Pc54* differential had an IT of ‘1+’ to race NQBK, Ajay had an IT of ‘3+;’ to race MBTG and Kasztan had an IT reaction of ‘3’ or ‘4’ when challenged with *Pca* race BRBG-94. Although the seedling reaction of *Pc54* against the NQBK race was low for scoring purposes, the distribution among the RILs was 47% 0; and 42% 1+ (Fig 1), suggesting that this contrast was sufficient to distinguish *Pc96* carriers from non-carriers. In the LSU field nursery, *Pc54* flag leaves had medium sized uredia and were scored as S while *Pc96* had no uredia and were scored as R. All RILs resembled one parent or the other. Resistance in the Pc54 × Pc96 population fit a single gene segregation model in the seedling test (Table 1). When the Ajay × Pc96 population was tested under a single gene model, it did not fit the ratio and likely segregated for an additional *Pc* gene detectable using the MBTG race. However, the linkage analysis used in this study is expected to be robust to multiple genes segregating in a population, making no assumption about the number of genes or the size of their effect. Segregation in the Pc96 × Kasztan F_2_ population fit a single dominant gene model, but the F_2:3_ population did not fit this model (Table 1). Given the fit with the expected one gene ratio using F_2_ plant phenotypes we hypothesize that this is a false negative result that could be due to greater than expected heterozygosity because of such factors as sampling error and/or plants escaping infection.

**Table 1:** Crown rust reaction segregation ratios in three biparental populations.

				Number of plants with phenotype						
Population	Generation	Test	Race	R^a^	Seg	S	Total	Model	χ^2^	*P* value
Pc54 × Pc96	F_5:6_	Seedling	NQBK	57	10	55	122	1:1	0.04	0.841 ns
Ajay × Pc96	F_2:3_	Seedling	MBTG	82	52	5	139	1:2:1	94.12	0.00001
Pc96 × Kasztan	F_2_	Seedling	BRBG-94	128		41	169	3:1	0.04	0.824 ns
	F_2:3_	Seedling	BRBG-94	31	114	23	168	1:2:1	22.19	0.000015

^a^Lines were rated as resistant if they had seedling IT below "3". All lines with ratings above these values were designated as susceptible; **R**: Resistant; **Seg**: Segregating; **S**: Susceptible.

### Linkage analysis

#### Pc54 *×* Pc96 seedling response

**Fig 2:**
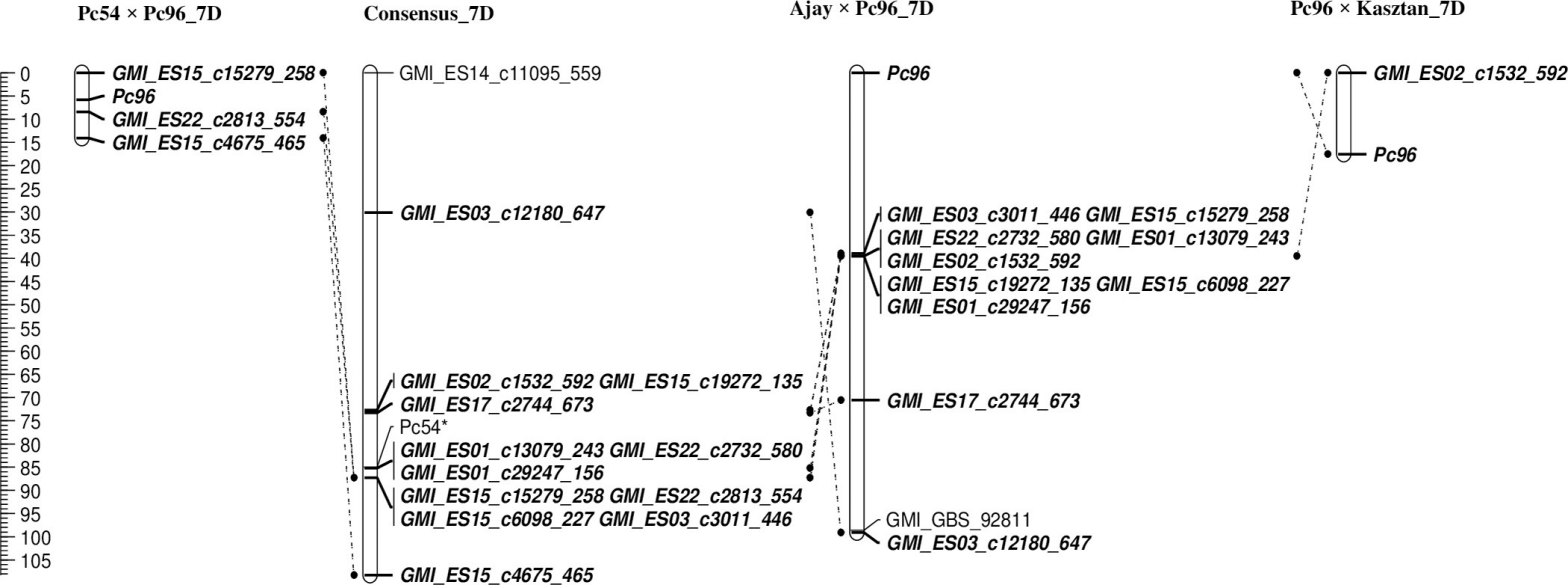
Linkage maps showing the location of the *Pc96* crown rust resistance gene. “Consensus Map” refers to the chromosome 7D consensus genetic map of Chaffin et al. [46]. The ruler scale on the left indicates map distances in centiMorgans (cM). * The estimated position of *Pc54* on consensus map [19].

The 587 segregating SNPs were assigned to 21 linkage groups (Table 2). The total genetic distance across all groups was 3267.94 cM and *Pc96* was mapped to chromosome 7D flanked by GMI_ES15_c15279_258 and GMI_ES22_c2813_554 with 5.8 cM and 2.6 cM between these loci and *Pc96*, respectively (Fig 2). Another SNP closely linked to *Pc96* was GMI_ES15_c4675_465 (8.3 cM).

**Table 2:** Number of polymorphic SNPs between parental lines and mapped SNPs in the two oat mapping populations.

Population	SNP No. (polymorphic)	SNP No. (mapped)	Coverage (cM)
Pc54 ***×*** Pc96	726	587	3267.94
Ajay ***×*** Pc96	630	518	5147.47

#### Ajay × Pc96

To validate the chromosomal location of *Pc96*, 139 lines from the biparental Ajay × Pc96 population were used. The 518 polymorphic markers were assigned to 22 linkage groups through automated hierarchal clustering and the total genetic distance was 5147.47 cM (Table 2). Consistent with the phenotype segregation results, two QTL were detected in the Ajay × Pc96 population. LOD scores greater than the threshold (3.0 and 3.4) were observed on chromosome 6C and 7D. Markers closest to the peak were GMI_ES14_c7250_379 on chromosome 6C and GMI_ES02_c1532_592 on chromosome 7D and these two markers were used to make two sub-populations for linkage mapping of resistance genes (as described above). Linkage analysis placed what we presume is the *Pc96* gene on chromosome 7D 39 cM distal to the SNP markers GMI_ES15_c15279_258 and GMI_ES03_c3011_446 (Fig 2). These SNPs are both located at 87.3 cM on the consensus map. Six other SNPs were not tightly linked to *Pc96*.

A second locus contributed by the *Pc96* differential was detected in the Ajay × Pc96 population on chromosome 6C. This gene, called *Pc_MBTG_6C*, was loosely linked (34.5 cM distal) to the SNP GMI_ES14_c7250_379 which corresponds to 75.5 cM on the chromosome 6C consensus map.

#### Pc96 × Kasztan

SNP assays were designed for seven markers and run on four parents: Pc96, Pc54, Ajay and Kasztan to identify those with potential to validate *Pc96* map location in the Pc96 x Kasztan population. Six markers were amplified during PACE PCR assay for different parental lines. Only one marker, GMI_ES02_c1532_592, was segregating in Pc96 × Kasztan population (S2 Table). Validation of the *Pc96* map location in the Pc96 × Kasztan population was performed using a PACE PCR assay developed from the flanking sequence of SNP GMI_ES02_c1532_592 (details of assay development results presented in S2 Table). *Pc96* was 17.5 cM from GMI_ES02_c1532_592 (Fig 2).

#### Pc54 *×* Pc96 field response

**Fig 3:**
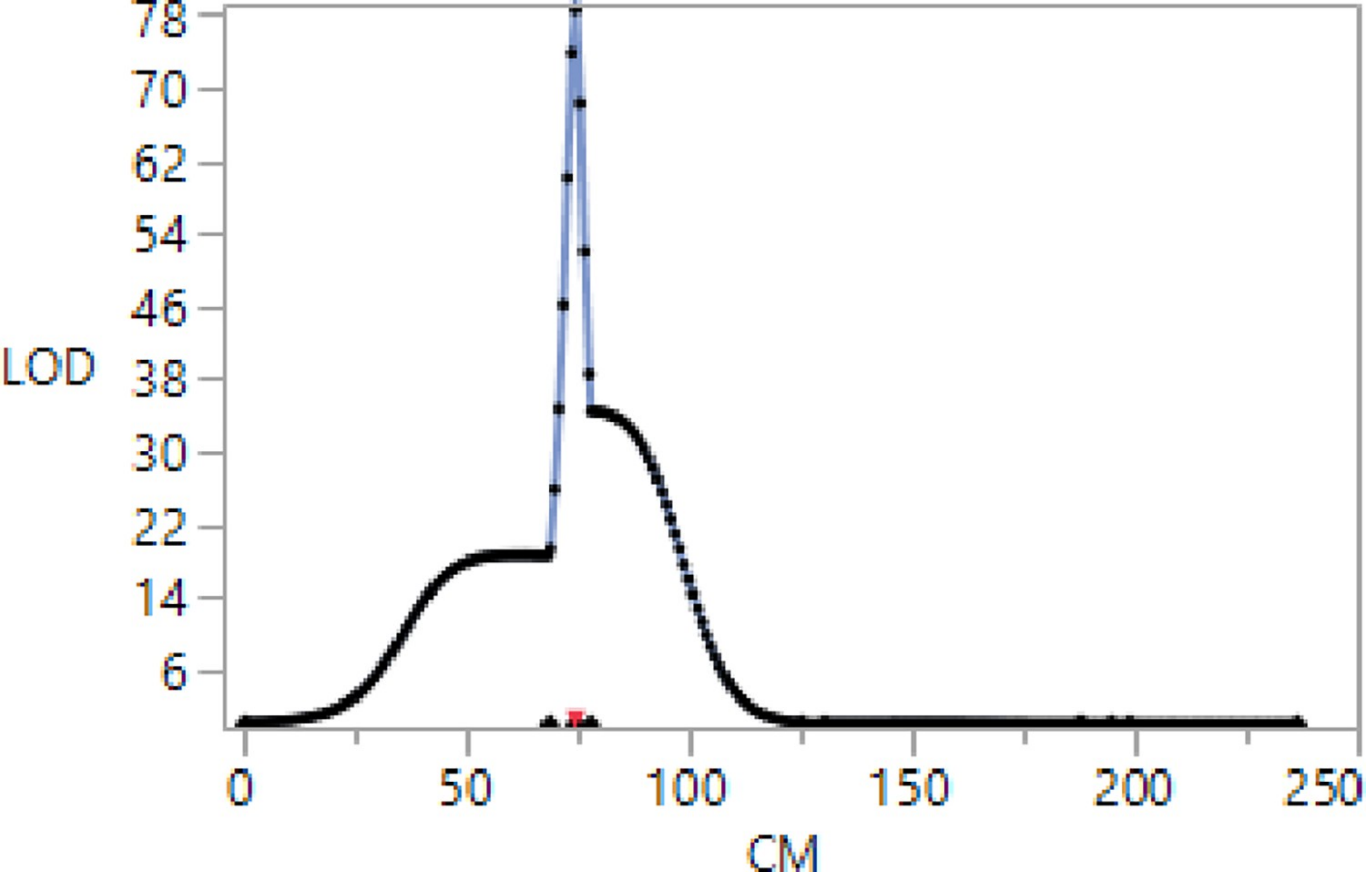
LOD profile generated for field response to crown rust infection from a Pc54 × Pc96 F5-derived oat population on chromosome 7D where each SNP marker is represented by a single point.

Field response data of Pc54 × Pc96 RILs were consistent with a 1:1 segregation ratio determined by a single resistance gene (Fig 1). The Peak LOD score was 78.62 at 87.3 cM on chromosome 7D with R^2^ = 0.93 and the additive effect of the Pc96 differential line allele was 0.98 (Fig 3).

### SNP performance in elite germplasm

In order to estimate allele frequencies in the wider population of cultivated oat, genotype data for SNPs within the chromosomal region most likely to contain the *Pc96* gene was examined. Fourteen markers were segregating within 48–91 cM of *Pc96* in three populations (Fig 2 and S4 Table). Most of the markers aligned within the 72 to 87 cM region of chromosome 7D on the consensus map. Data was also examined for additional markers within that region of the consensus map. One-hundred-forty-four lines, including 30 *Pc* differential lines, presumed to not carry the *Pc96* gene were compared for genotype at nine randomly selected SNPs located on chromosome 7D between 72.7 to 87.3 cM for which the *Pc96* differential was observed to carry the rare allele (S3 Table). SNPs were found to misclassify between 16 to 21% of *Pc96* non-carriers. However, the genotype data of GMI_ES05_c14633_290 in combination with that of any of the other six SNPs at 87.3 cM evaluated in this study would reduce the false positive rate to a more reasonable 5%. Combined genotype data for GMI_ES05_c14633_290 and GMI_ES01_c8043_192 resulted in a 2.7% false positive rate. A four-marker haplotype consisting of GMI_ES05_c14633_290 (72.7 cM), GMI_ES01_c8043_192 (85.2 cM), GMI_ES03_c3828_733 (85.2 cM), and any one of the six evaluated SNPs at 87.3 cM correctly classified all non-carriers of Pc96 except three (Mortlock, PI263412-1 and Red Algerian) resulting in a false positive rate of 2.1% (S3 Table).

## Discussion

Discussion of the locations of these resistance genes below are always in terms of cM locations on the consensus map Chaffin et al. [46] (Fig 2 and S4 Table). In this study we determined the genetic location for *Pc96* in a RIL population and validated the map position in two additional populations. Based on this, *Pc96* is on chromosome 7D between 48.3 to 91.2 cM on the consensus linkage map of Chaffin et al. [46] with the most probable location between 72.7 and 87.3 cM. Linkage analysis in the Pc54 × Pc96 population placed *Pc96* to within an 8.4 cM window, while that in the Ajay × Pc96 population was only able to place the gene at 39 cM from two markers. Disappointingly, the single segregating marker evaluated in the Pc96 × Kasztan population was also only loosely linked. These maps do, however agree in the general placement of *Pc96* to the broad window of chromosome 7D and are generally consistent with placement to approximately 87.3 cM on the consensus linkage map of Chaffin et al. [46]. Additionally, markers closely linked with *Pc96* were Blasted against the (“*Avena sativa*–PepsiCo v1/Consensus_2018/Infinium 2020, https://oat.triticeaetoolbox.org/”) and were placed to the 7D chromosome between 460 and 490 Mbp (S4 Table).

Other investigations have identified *Pc* genes in this chromosomal region. Evidence of linkage in the broader *Pc96* gene region was reported for *Pc35* [32], *Pc38* (chromosome 9D) [12], and *Pc54* [19]. Segregation analysis has linked *Pc38* with *Pc62* and *Pc63* [47]. On the same linkage group, but at least 30 cM proximal to *Pc96* are also *Pc58a* [20, 21], and *QPc*.*CORE*.*02* [37]. *PcA*, later assigned the gene symbol *Pc96* [32], was found to be linked to *Pc35*, a gene derived from *A*. *sterilis* [48]. These two genes came from different sources, so they should be linked in repulsion. This suggests that combination within a single line would be rare, but a precise map location for *Pc35* remains to be identified. The crown rust resistance gene *Pc38* was originally identified in *A*. *sterilis* [11, 49], and mapped with restriction fragment length polymorphism (RFLP) markers [12]. *Pc38* mapped to a region that corresponds to the Mrg02 consensus map linkage group between 73.3 and 118.5 cM. This position of *Pc38* overlaps with the most probable location of *Pc96*, and the recombination between *Pc38* and *Pc96* could be evaluated if this combination of genes is desired. Yimer et al. [19] mapped *Pc54* to the same location as *Pc96*, in part using the Pc54 × Pc96 population analyzed in this study. A comparison of Pc54 × Pc96 phenotypes from growth chamber screening with NQBK (avirulent on *Pc96*) and LLMG (avirulent on *Pc54*) [19] indicate that these two genes are linked rather than allelic. Crossover events produced 2 RILs which were homozygous carriers of both genes and only four RILs carried the susceptible alleles for both *Pc54* and *Pc96* (S5 Table). Linkage analysis with carrier status of *Pc54* and *Pc96* both coded as markers indicated that *Pc54* was proximal to *Pc96* (Fig 2) [19].

Of the *Pc58* complex genes, *Pc58a* and *Pc58c* are positioned at 10.8 cM [50] on the consensus map which is more than 50 cM from the QTL region of *Pc96*. Whereas *Pc58b* is at 110.4 cM [20, 21, 51]. *Pc96* is implausible as a *Pc58* cluster gene. Using association mapping methods Esvelt Klos et al. [37] reported the presence of the seedling resistance QTL *QPc*.*CORE*.*02* on linkage group Mrg02. This QTL was identified in the CORE collection of elite oat lines and influenced variation in crown rust disease response in field nurseries in Louisiana, North Dakota, and Manitoba in 2010 and 2011. *QPc*.*CORE*.*02* was best represented by the SNP GMI_GBS_94371 at 28.1cM, at least 30 cM proximal to *Pc96*, suggesting that *QPc*.*CORE*.*02* is unlikely to represent the effect of the *Pc96* gene. It seems that chromosome 7D contains multiple *Pc* genes (*Pc35*, *Pc38*, *Pc54*, *Pc58a*, *Pc62*, *Pc63* & *Pc96*). These characteristics make chromosome 7D a promising target for further investigation. For example, the map positions of *Pc62* and *Pc63* are still only roughly defined.

The mapping crosses with the Pc96 differential were made under the assumption that this line carried a single gene conferring seedling resistance to crown rust. Interestingly, an additional gene, also originating from the *Pc96* differential line, with a perceptible affect appeared to be segregating in the Ajay × Pc96 population. This was originally suggested by the lack of fit to a single segregating gene model (Table 1). In addition to the QTL on chromosome 7D, presumed to be *Pc96*, a second QTL on chromosome 6C was resolved with linkage mapping to an unknown gene, *Pc_MBTG_6C*, on chromosome 6C. Few chromosome 6C markers were segregating in the Ajay x Pc96 population and linkage mapping was only able to detect loose linkage (34.5 cM in the population-specific map) between *Pc_MBTG_6C* and a single SNP with location corresponding to 110.4 cM on the consensus map. This corresponds to report of a minor QTL, QPc.APR-6C, on chromosome 6C at 625 Mb [52]. Seedling resistance was used to map the resistance locus in this study whereas field data was used to identify the APR type resistance in the Nazareno [52] study. It is not unusual for a *Pc* differential line to be found to be segregating for multiple crown rust resistance genes. For example, the Pc50 differential line has been found to carry *Pc50-2* [53], *Pc50-4* [54] and *Pc50-5* [17]. The Pc58 differential line carries three genes conferring seedling stage resistance (*Pc58a*, *Pc58b* and *Pc58c*) and several genes conferring quantitative types of resistance [20, 21, 50]. Many of the oat *Pc* gene differential lines currently available were developed by phenotypic selection which would allow additional unobserved genes to remain in the line during development.

Markers identified in this study linked to the *Pc96* gene could be of interest to oat breeders for use in marker assisted selection (MAS). The complications inherent to identifying markers suitable to MAS in a new cross are illustrated by our attempts to characterize inheritance of *Pc96* in the Pc96 × Kasztan population (S2 Table). Although we began the PACE PCR assay design process with 7 SNPs of the successfully designed assays only a single linked marker was segregating in the population. Many additional assays would need to be designed to obtain working assays for two segregating markers that bracket the desired gene with tighter linkage than here obtained.

Markers identified in this study linked to the *Pc96* gene could also be of interest to oat breeders and pathologists for use in attributing unknown crown rust resistance to the presence of *Pc96*. Markers used for this purpose must not only be linked to the gene of interest but should ideally be specific to the unique haplotype that surrounds the gene. SNPs that are present in other haplotypes within the population will mis-classify germplasm as carriers or non-carriers to some extent. We evaluated the ability of markers in the *Pc96* gene region to correctly classify susceptible germplasm and other *Pc* differential lines as non-carriers of the *Pc96* gene. The nine SNPs we evaluated all mis-classified some non-carrier lines as carriers with a false positive rate up to 21% (S3 Table). This is too high to qualify these SNPs as potential diagnostic markers for *Pc96*. Prediction combining GMI_ES05_c14633_290 and GMI_ES01_c8043_192 reduced the false positive rate to 2.7%. These markers also flank the *Pc96* locus. Further investigation will be required to identify markers unique to the haplotype carrying the *Pc96* gene and capable of unambiguously diagnosing carrier status in unrelated germplasm.

Historical reports of the effectiveness of *Pc96* in producing an effective level of resistance to naturally occurring crown rust have been encouraging. Chong and Brown [32] found that this gene was successful in controlling more than 97% of oat crown rust isolates collected from North American regions (US & Canada) during 1991 to 1994. Menzies et al. [33] observed 95% of all isolates of *Pca* from Canada to have avirulence to *Pc96* during 2010 to 2015. In Eastern Europe this gene was also found effective against East European oat crown rust pathotypes. In 2006, *Pc96* was classified in Eastern Europe as a very efficient source of resistance, with a value of resistance efficiency score 0.857 [54]. The efficiency of this gene in Poland in 2017–2019 exceeded 95% [55] and was even higher than reported in 2013–2015 when about 20% of analyzed isolates broke down the resistance determined by this gene [56]. Within the Matt Moore Buckthorn Nursery at St. Paul, MN, the *Pc96* differential line exhibited a consistent level of moderate resistance in 2016, 2019, and 2020 as indicated by severity ratings of 23%, 25%, and 20%, respectively (Tyler Gordon, unpublished). Given that the frequency of virulence remains low in natural populations, it may be beneficial to use *Pc96* in oat breeding for crown rust resistance. It has been previously suggested that *Pc96* is a good candidate gene for combining with other effective *Pc* genes including *Pc42*, *Pc45*, *Pc48*, *Pc50*, *Pc62*, *Pc68*, and *Pc91* [29, 57, 58]. In addition to the moderate effectiveness of *Pc96* gene in the Matt Moore Buckthorn Nursery, we also observed moderate effectiveness of genes *Pc58*, *Pc64*, *Pc91* and *Pc94*. In a recent report of three years data (2016, 2019 and 2020), lines carrying those *Pc* genes had 19–32% rust severity in the field (Tyler Gordon, unpublished). This information suggests potential for utilization in combination with *Pc96*.

In conclusion, we mapped *Pc96*, which confers race-specific crown rust resistance in oat, to a region which corresponds to the oat consensus linkage group Mrg02 (chromosome 7D) approximately at 87.3 cM. This increases the number of crown rust seedling resistance genes placed to the oat genome in the context of modern high-throughput molecular markers. This study also showed that the Pc96 differential line was segregating for another *Pc* gene on chromosome 6C.

## Supporting information

S1 Table The race designation and virulence pattern against 17 *Pc* gene differential lines for the *Puccinia coronata* f.sp. *avenae* isolates used in this study. (XLSX)

S2 Table Marker alleles of 4 oat parental lines and Pc96 × Kasztan population genotyped with the six polymorphic PACE markers designed from the respective SNP sequences. (XLSX)

S3 Table Genotype cluster calls at nine SNPs on chromosome 7D at 87.3 cM for non-carriers of Pc96 compared with the Pc96 differential line. (XLSX)

S4 Table SNP markers closely linked with *Pc96* crown rust resistance on chromosome 7D in three populations indicating position on de novo and the consensus map of Chaffin et al. [46] and physical position on the PepsiCo v1a/OT3098v2. (XLSX)

S5 Table The number of resistant and susceptible Pc54 × Pc96 RILs evaluated after inoculation with two races of *Puccinia coronata* f. sp. *avenae*. (XLSX)
